# Effects of Protein Levels of Diets and Yeast Culture Supplementation on Performance, Antioxidant Capacity, and Intestinal Health in Laying Hens

**DOI:** 10.3390/microorganisms14102223

**Published:** 2026-10-02

**Authors:** Siyu Chen, Keying Zhang, Jianping Wang, Shiping Bai, Qiufeng Zeng, Wenpan Wang, Liang Wang, Penghui Yang, Yue Xuan, Xuemei Ding

**Affiliations:** 1 Key Laboratory of Animal Disease-Resistance Nutrition, Ministry of Agriculture and Rural Affairs, Key Laboratory of Sichuan Province, Animal Nutrition Institute, Sichuan Agricultural University, Chengdu 611130, China; 2 Shaanxi Xinhanbao Biotechnology Co., Ltd., Xianyang 712000, China

**Keywords:** yeast culture, laying hens, production performance, antioxidant capacity, intestinal health

## Abstract

Yeast culture (YC) can improve laying hen performance, but whether this effect depends on dietary protein level remains unclear. This experiment investigated the effects of adding YC to diets with different protein levels on the performance and intestinal health of laying hens. A total of 504 Lohmann pink laying hens aged 55 weeks were selected and randomly assigned to 4 treatments, with 7 replicates per treatment and 18 hens per replicate. A 2 × 2 factorial design was used, with two CP levels of 15.5% (NP) and 14.5% (LCP), and two YC supplementation levels of 0% and 1%. The feeding trial lasted 12 weeks. YC significantly increased the laying rate (*p* < 0.05). CP and YC interacted to affect the laying rate (*p* < 0.05). YC significantly increased serum CAT and GSH-Px activities, reduced PCO, and increased trypsin activity (*p* < 0.05). CP and YC interacted to affect GSH-Px and trypsin activities (*p* < 0.05). GSH-Px and trypsin activities were significantly higher in the LCP-YC group than in the LCP-0 group (*p* < 0.05). YC significantly increased jejunal villus height (VH) (*p* < 0.05). A significant CP × YC interaction was observed for jejunal VH; supplementing the LCP diet with YC significantly increased VH (*p* < 0.05). YC significantly increased the percentage of goblet cells in the jejunum, the contents of IgG and IgM in the jejunal mucosa, and decreased TNF-α content (*p* < 0.05). IFN-γ content was also significantly lower in the LCP-YC group than in the LCP-0 group (*p* < 0.05). YC affected cecal microbial α-diversity, while CP and YC interacted to affect β-diversity and the relative abundance of certain bacterial taxa (*p* < 0.05). In conclusion, under these experimental conditions, YC improves the laying rate and antioxidant capacity and supports intestinal health in laying hens. A CP × YC interaction was observed, but the apparently stronger response to YC in the LCP group should be interpreted cautiously because the LCP-0 and LCP-YC diets differed in analyzed calcium concentration.

## 1. Introduction

In large-scale poultry farming, sustained high production efficiency often causes problems such as intestinal damage, immune imbalance, and gut microbiota disruption as laying hens age [1]. In addition, hens in the late laying phase experience reduced ovarian antioxidant capacity, fewer and poorer-quality oocytes, and less stable yolk deposition [2,3,4]. As the physiological functions of laying hens decline, laying performance and egg quality deteriorate significantly, which reduces economic returns. Therefore, improving performance in late-stage laying hens, delaying aging, and extending the laying cycle through appropriate feed formulation and nutritional management are key strategies for sustaining production.

Yeast culture (**YC**) is a complex and nutrient-rich microbial product obtained by fermenting yeast in specific culture media [5]. It contains yeast cells, fermentation metabolites, and modified culture media, including β-glucans, mannan, proteins, peptides, vitamins, minerals, enzymes, and various unidentified growth factors [6]. In laying hens, dietary YC supplementation significantly improves performance [7]. In breeding flocks, yeast bioactives significantly increase immunoglobulin A (**IgA**) levels in hatching egg yolks, improve intestinal villus development in chicks, and support chick performance and intestinal health [8]. In broiler chickens, 0.1% YC can replace antibiotics to improve performance, and yeast nucleotides can alleviate the inhibitory effects of coccidia on villus development [9,10]. In geese, supplementation with 2% YC significantly increases antioxidant levels and digestive enzyme activity, and promotes growth [11].

Low-protein diets have attracted increasing interest because they reduce reliance on high-quality protein sources, lower feed costs, and decrease nitrogen emissions. However, reducing dietary protein levels may also alter the metabolism of other nutrients, potentially compromising animal health and performance [12]. Adding YC to low-protein diets is an effective way to address this problem [13]. YC is also a high-quality protein source, and high inclusion levels may improve nutrient digestion and absorption by favorably altering the gut microbial community. Despite growing interest in YC as a growth promoter, few studies have examined the interaction between dietary crude protein (**CP**) levels and YC supplementation. We hypothesized that CP levels and YC supplementation would affect performance, antioxidant capacity, and intestinal health in hens in the late phase of production, and that the response to YC might depend on dietary CP levels. Therefore, this study used a 2 × 2 factorial arrangement to examine the effects of dietary CP levels, YC supplementation, and their interaction on the performance, antioxidant capacity, and intestinal health of laying hens in late production.

## 2. Materials and Methods

### 2.1. Animal Ethics Statement

The experimental protocol underwent ethical review and was approved by the Institutional Animal Care and Use Committee of Sichuan Agricultural University (Ethics Approval Code: SICAUAC 20221116004; Chengdu, China).

### 2.2. Experimental Animals and Design

For this experiment, 504 Lohmann pink laying hens aged 55 weeks were selected and randomly assigned to 4 treatments, with 7 replicates per treatment and 18 hens per replicate (6 layers/cage, 3 adjacent cages/replicate). Four adjacent replicate units formed one block, and each treatment occurred once per block, with treatment order randomized within each block. A 2 × 2 factorial design was used, with two CP levels of 15.5% and 14.5%, and two YC supplementation levels of 0% and 1%. The YC used in this study was produced by Shaanxi Xinhanbao Biotechnology Co., Ltd. (Xianyang, China) (yeast strain: *Saccharomyces cerevisiae* CICC1794; product name: Poultry Safety; fermentation substrate: corn, cottonseed meal, wheat bran; production process: yeast is cultured by liquid fermentation, then inoculated into a solid medium for anaerobic fermentation, and finally dried to produce the finished product; physical form: powder; storage conditions: store in a cool, dry place at room temperature; the quantity of live yeast cells is unknown). A corn-soybean meal-based diet was used, with its composition based on the China National Feeding Standard for laying hens [14]. All experimental diets were formulated to contain similar levels of metabolizable energy and essential amino acids. Diet composition and nutrient levels are presented in Table 1. Representative samples of each diet were analyzed in duplicate according to AOAC (2005) procedures [15]. Crude protein was analyzed using the Kjeldahl method (method 984.13). Calcium and phosphorus were determined after acid digestion using spectrophotometric methods (methods 968.08 and 965.17). The experiment took place at the Zitong base of Sichuan Sundaily Farm Ecological Food Co., Ltd. (Mianyang, China). The chicken house temperature was maintained at 22 ± 1 °C, relative humidity at 40 ± 10%, and artificial lighting was supplied for 16 h daily. The hens were vaccinated according to the standard immunization schedule. Throughout the trial, the hens had free access to feed and water. They received only powdered feed during the experiment. The first feeding was at 8:30 a.m., followed by an inspection of the housing and hens for abnormalities. The second feeding was at 3:00 p.m., followed by a further inspection of the housing and hens, egg collection, and recording of egg weight. Performance was assessed using each replicate as the experimental unit. The housing was cleaned regularly. Before the trial, all hens received a corn-soybean meal basal diet during a 2-week pre-feeding period, and laying rate was recorded. Hens were assigned to treatment groups according to laying rate to ensure no differences between groups. The feeding trial lasted 12 weeks.

### 2.3. Sample Collection

At the end of the experiment, one hen per replicate (selected based on proximity to mean body weight) underwent jugular venipuncture. Blood was divided among three collection tubes. After the tubes had been left to stand, the blood was centrifuged at 2126× *g* for 10 min. The serum supernatant was transferred to sterile Eppendorf tubes as serum samples, which were stored at −20 °C. After slaughter by cervical dislocation, the pancreas was collected and stored at −20 °C for measurement of pancreatic digestive enzyme activity, and cecal chyme was collected and stored at −80 °C for microbiological analysis. Jejunal and ileal contents were flushed out with pre-chilled sterile saline. A 1-cm segment from the middle portion was fixed in 4% paraformaldehyde for examination of intestinal morphology. The remaining jejunum was opened, and residual saline was gently blotted with sterile filter paper. The mucosa was scraped onto a microscope slide and transferred to a cryovial for storage at −80 °C until measurement of mucosal immune markers. Thus, all samples used to assess serum antioxidant capacity, pancreatic digestive enzyme activity, intestinal histology, jejunal immunity and inflammatory status, and cecal microbiota came from the same hen within each replicate.

### 2.4. Laying Performance

Throughout the experiment, the number of eggs laid, egg weight, feed intake, number of dirty eggs, number of broken eggs, and number of qualified eggs were recorded daily for each replicate. At the end of the experiment, data for weeks 1–6 and weeks 7–12 were aggregated, and the following measures were calculated for each replicate: laying rate, average daily feed intake (**ADFI**), average egg weight, daily egg weight, feed-to-egg ratio (**FCR**), and qualified egg rate. Here, FCR was defined as total feed intake (g) divided by total egg weight (g), and daily egg weight was defined as total egg weight (g) divided by the product of the number of laying hens and feeding days.

### 2.5. Egg Quality

Egg quality was assessed at week 12 of the trial, using 3 eggs from each replicate, and the mean of the 3 eggs represented that replicate. Albumen height, yolk color, and Haugh unit were determined with a multifunctional automatic egg quality analyzer (EMT-5200, Robotmation, Tokyo, Japan); eggshell strength was determined with an eggshell strength tester (EFG-0503, Bulader, Tokyo, Japan); eggshell thickness and egg shape index were measured with a vernier caliper. The egg shape index (ESI) was calculated as the ratio of egg length to egg width (ESI = L/W), according to the Chinese Agricultural Industry Standard [16].

### 2.6. Serum Antioxidant Capacity

At the end of the experiment, blood was collected from the jugular vein of one hen per replicate. Total antioxidant capacity (**T-AOC**), glutathione peroxidase (**GSH-Px**) activity, superoxide dismutase (**SOD**) activity, catalase (**CAT**) activity, malondialdehyde (**MDA**) content, and protein carbonyl content (**PCO**) in hen serum were measured with commercial kits (Nanjing Jiancheng Bioengineering Institute, Nanjing, China). The results were read using the Microplate Reader Infinite SpectraMax 190 (Molecular Devices, Silicon Valley, CA, USA). Assays were performed according to the kit instructions and previously published methods [17].

### 2.7. Pancreatic Digestive Enzyme Activity

At the end of the experiment, one hen was selected per replicate for blood collection, after which it was slaughtered, and its pancreas was collected. Pancreatic samples were suspended in saline at a weight (g) to volume (mL) ratio of 1:9, homogenized, and centrifuged at 2126× *g* for 10 min; the resulting supernatant was collected for analysis. The activities of amylase, lipase, chymotrypsin, and trypsin were determined using commercial kits (Nanjing Jiancheng Bioengineering Institute, Nanjing, China). Total protein concentration was measured by the Bradford assay. Assays followed the kit instructions and previously published methods [18].

### 2.8. Intestinal Histological Analysis

At the end of the experiment, one hen was selected per replicate for blood collection; it was then slaughtered, and tissue samples were collected from the jejunum and ileum. Paraformaldehyde-fixed intestinal samples were embedded in paraffin and cut into 5-μm-thick transverse sections; these were stained with hematoxylin and eosin (H&E). Villi were photographed under a microscope, and the height of 8 consecutive villi (**VH**, μm) and crypt depth (**CD**, μm) in each sample were measured with Image-Pro Plus 6.0. The ratio of villus height to crypt depth (**V/C**) was then calculated for each villus. The measurements of the 8 villi were then averaged to obtain a single value for each replicate. The observer was blinded to treatment, selecting villi that were intact, upright, uniform in morphology, and relatively tall, with intact crypt and muscular layer structures. The same observer performed all assessments.

### 2.9. Jejunal Immunity and Inflammatory Status

At the end of the experiment, one hen was selected per replicate for blood collection; the hen was then slaughtered, and jejunal tissue was collected and its mucosa scraped. Jejunal paraffin sections were stained using the periodic acid-Schiff (PAS) staining method. Goblet cells were examined and photographed under a 400× objective on a BX51 microscope (Olympus, Tokyo, Japan). Six villi with clearly defined morphology were selected, and the number of goblet cells per 100 epithelial cells was calculated for each villus. Subsequently, the percentage of goblet cells on the six villi was averaged to obtain a single value for each replicate.

Jejunal mucosal samples were suspended in saline at a mass (g) to volume (mL) ratio of 1:9, homogenized, and centrifuged at 2126× *g* for 20 min; the resulting supernatant was collected for analysis. Enzyme-linked immunosorbent assay (ELISA) kits (Jiangsu Meimian Industrial Co., Ltd., Yancheng, China) were used to measure immunoglobulin G (**IgG**), immunoglobulin M (**IgM**), secretory immunoglobulin A (**sIgA**), interferon-γ (**IFN-γ**), and tumor necrosis factor-α (**TNF-α**) levels. These measurements were performed according to the kit instructions and the methods described in previous reports [19].

### 2.10. Cecal Microbiota

One cecal content sample was obtained from each replicate (*n* = 7 per treatment group) for microbiota analysis, and samples were submitted to Luoning Biotechnology Co., Ltd. (Chengdu, China), for sequencing. The following procedure was used. Sample DNA purification: genomic DNA (gDNA) was extracted and purified with the BIOMICS DNA Microprep Kit (Zymo Research, Irvine, CA, USA). gDNA integrity was checked by 0.8% agarose gel electrophoresis, and nucleic acid concentration was measured with a Tecan F200 (PicoGreen dye method). PCR amplification: For the selected sequencing region, the V4 region of the 16S rRNA gene in the samples was amplified with primers 515F (5’-GTGYCAGCMGCCGCGGTAA-3’) and 806R (5’-GGACTACHVGGGTWTCTAAT-3’). PCR product detection, purification, and quantification: Target fragments in the PCR products were detected by agarose gel electrophoresis. Suitable target bands were recovered and purified with the Zymoclean Gel Recovery Kit (Zymo Research, Irvine, CA, USA), quantified with the Qubit^®^ 2.0 Fluorometer (Thermo Fisher Scientific, Waltham, MA, USA), and pooled in equimolar amounts. Library construction: Libraries were prepared with the MGIEasy Universal DNA Library Prep Kit (MGI Tech Co., Ltd., Shenzhen, China). High-throughput sequencing: Sequencing was conducted on the MGI DNBSEQ-G99RS platform, with the DNBSEQ-G99RS high-throughput sequencing reagent kit (MGI Tech Co., Ltd., Shenzhen, China) serving as the sequencing reagent, in PE250 mode. Data analysis: Paired-end sequences were assembled using FLASH, and quality filtering was performed using QIIME2. Chimeric sequences were removed using the UCHIME algorithm. Sequence clustering was performed using UPARSE to generate operational taxonomic units (OTUs) at a similarity threshold of ≥ 97%.

### 2.11. Statistical Analysis

The Shapiro–Wilk and Levene’s tests were used to assess data normality and homogeneity of variances, respectively. All analyses were performed using SAS 9.2 statistical software (SAS Institute, Cary, NC, USA). Laying performance data for weeks 1–6 and 7–12 were analyzed using a linear mixed model with the MIXED procedure and restricted maximum likelihood estimation. Dietary CP level, YC, time, and all two- and three-way interactions were included as fixed effects. Block was included as a random effect to account for the randomized complete block design, and replicate was specified as the repeated subject. The model was: *Y_ijkl_* = *µ* + *B_k_* + CP*_i_* + YC*_j_* + *T_l_* + (CP × YC)*_ij_* + (CP × T)*_il_* + (YC × T)*_jl_* + (CP × YC × *T*)*_ijl_* + *ε_ijkl_*, where *Y_ijkl_* is the dependent variable; *µ* is the overall mean; *B_k_* is the random effect of block; CP*_i_*, YC*_j_*, and *T_l_* are the fixed effects of dietary CP level, YC, and time, respectively; the interaction terms represent the corresponding two- and three-way effects; and *ε_ijkl_* is the residual error. The repeated measurements were modeled with replicate as the subject. Compound symmetry (CS), first-order autoregressive [AR(1)], and unstructured (UN) covariance structures were compared using AIC and BIC. Because only two equally spaced time points were analyzed, the CS and AR(1) structures are mathematically equivalent; CS was selected for parsimony. Block was retained as a random effect regardless of its estimated variance to reflect the experimental randomization. Type III tests of fixed effects were performed using Kenward–Roger denominator degrees of freedom. Least-squares means were compared using the PDIFF option with Tukey’s adjustment. When an interaction was significant, simple effects were evaluated within the relevant levels of the interacting factors. Statistical significance and a tendency were defined as *p* ≤ 0.05 and 0.05 < *p* ≤ 0.10, respectively. For response variables measured at only one time point, including egg quality, serum antioxidant capacity, pancreatic digestive enzyme activities, intestinal mucosal structure, and immune and inflammatory status, no repeated-measures term was included. These variables were analyzed separately using a linear mixed model with CP, YC, and their interaction as fixed effects and block as a random effect: *Y_ijk_* = *µ* + *B_k_* + CP*_i_* + YC*_j_* + (CP × YC)*_ij_* + *ε_ijk_*. Results are presented as least-squares means and standard errors. Alpha diversity was estimated using four indices (Chao1, ACE, Shannon, and Simpson) in QIIME2. Beta diversity was assessed using Bray–Curtis distance matrices generated in QIIME2, followed by PCoA and hierarchical clustering. A factorial PERMANOVA (distance ~ CP + YC + CP: YC) was performed with 999 permutations using vegan::adonis2. Homogeneity of multivariate dispersion was tested using PERMDISP with 999 permutations (vegan::betadisper and permutest).

## 3. Results

### 3.1. Laying Performance

Table 2 presents the effects of YC on the laying performance of hens fed diets with different protein levels. Compared with the NP group, the LCP group had significantly lower average egg weight (*p* < 0.05). Compared with 0% YC, supplementation with 1% YC significantly increased the laying rate (*p* < 0.05). CP and YC also interacted to affect laying rate, ADFI, daily egg weight, and qualified egg rate (*p* < 0.05). Subsequent simple effects analysis indicated that, relative to the LCP-0 group, the LCP-YC group had a significantly higher laying rate (*p* < 0.05), significantly greater ADFI (*p* < 0.05), significantly greater daily egg weight (*p* < 0.05), and significantly higher qualified egg rate (*p* < 0.05).

### 3.2. Egg Quality

The effects of YC on egg quality at different dietary protein levels are shown in Table 3. Compared with the NP group, yolk color was significantly lower in the LCP group (*p* < 0.05). Compared with the 0% YC group, yolk color in the 1% YC group tended to improve numerically (*p* = 0.094). Neither CP nor YC significantly affected albumen height, Haugh unit, eggshell strength, eggshell thickness, or egg shape index (*p* > 0.05).

### 3.3. Serum Antioxidant Capacity

The effects of YC on serum antioxidant capacity in hens fed diets with different protein levels are shown in Table 4. Compared with 0% YC, supplementation with 1% YC significantly increased serum CAT and GSH-Px activities (*p* < 0.05), and significantly reduced PCO (*p* < 0.05). CP and YC also interacted to affect GSH-Px activity (*p* < 0.05). Subsequent simple effects analysis showed that, relative to the LCP-0 group, GSH-Px activity was significantly higher in the LCP-YC group (*p* < 0.05).

### 3.4. Pancreatic Digestive Enzyme Activity

The effects of YC on pancreatic digestive enzyme activities at different dietary protein levels are shown in Table 5. Compared with the NP group, trypsin activity was significantly higher in the LCP group (*p* < 0.05). Compared with 0% YC, supplementation with 1% YC significantly increased trypsin activity (*p* < 0.05) and decreased chymotrypsin activity (*p* < 0.05). CP and YC also interacted to affect trypsin activity (*p* < 0.05). Subsequent simple effects analysis showed that, relative to the NP-YC group, the LCP-YC group had significantly higher trypsin activity (*p* < 0.05).

### 3.5. Intestinal Mucosal Structure

**Figure 1:**
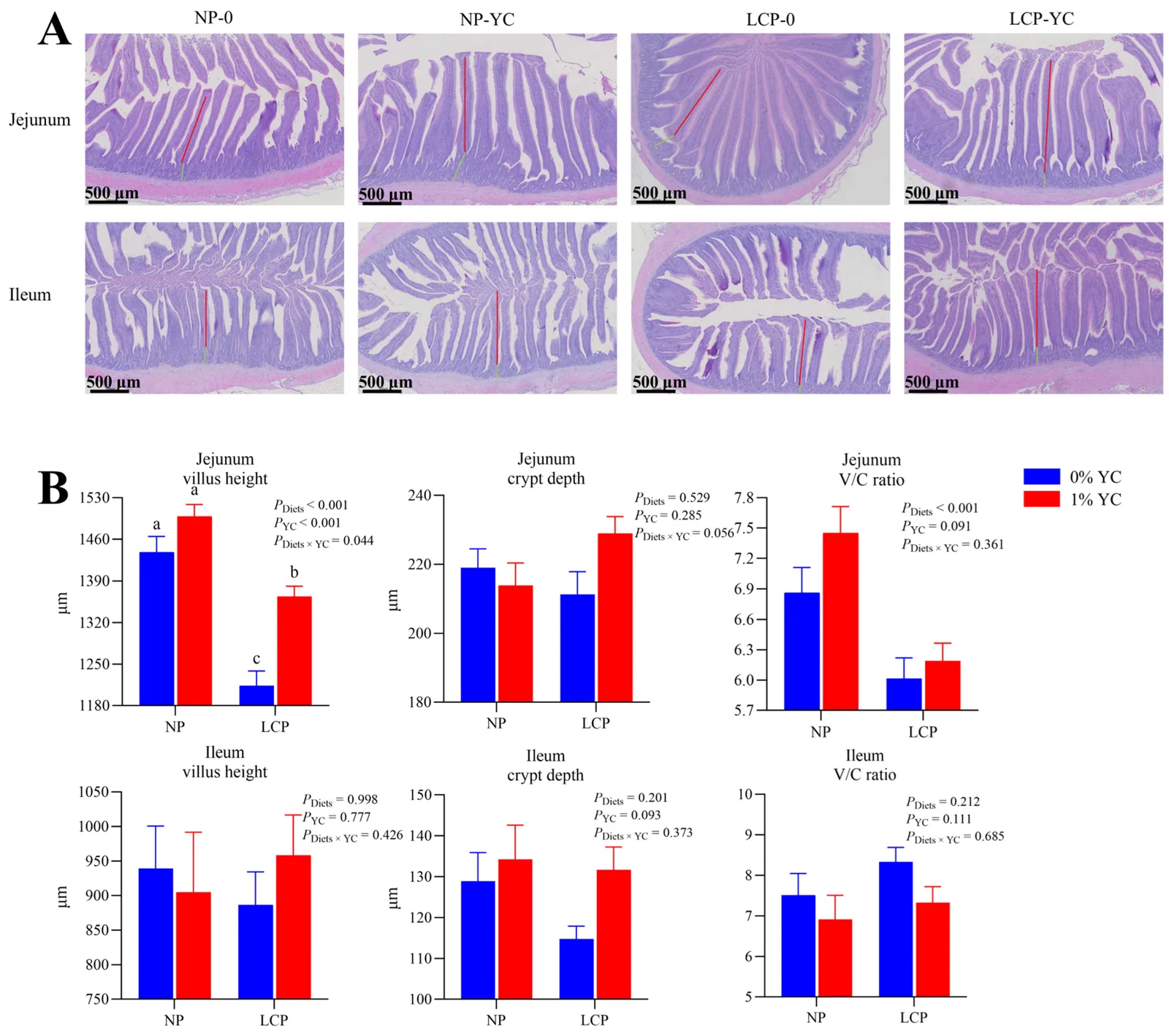
Effects of Yeast Culture on Intestinal Tissue Morphology of Laying Hens Fed Diets with Different Protein Levels. (**A**) Sections of the jejunum and ileum (H&E, 40×). Red lines indicate villus height, and green lines indicate intestinal crypt depth. (**B**) Villus height, crypt depth, and V/C ratio of the jejunum and ileum. V/C ratio = villus height/crypt depth. Data are expressed as least-squares means and standard errors (*n* = 7). Different letters indicate significant differences (*p* < 0.05), while the same letters indicate no significant difference between groups.

The effects of YC on intestinal morphology at different dietary protein levels are shown in Figure 1. Compared with the NP group, the LCP group had significantly lower jejunal VH and V/C ratio (*p* < 0.05), whereas CD did not change significantly (*p* > 0.05). Compared with 0% YC, supplementation with 1% YC significantly increased jejunal VH (*p* < 0.05), numerically increased the V/C ratio (*p* = 0.091), and did not significantly affect CD (*p* > 0.05). CP and YC also interacted to affect jejunal VH (*p* < 0.05). Subsequent simple effects analysis showed that, relative to the LCP-0 group, VH was significantly higher in the LCP-YC group (*p* < 0.05). Finally, YC had no significant effect on ileal morphology under different protein levels in the diet (*p* > 0.05).

### 3.6. Immune and Inflammatory Status

**Figure 2:**
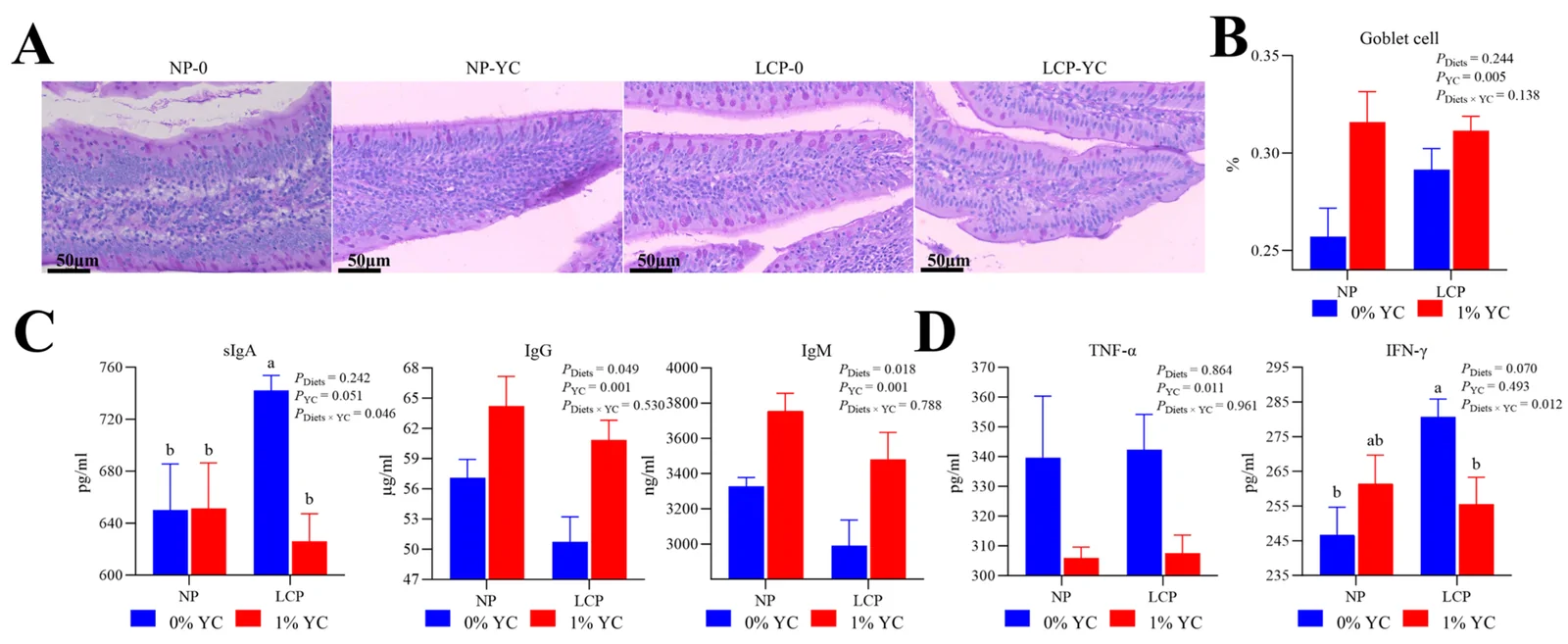
Effects of Yeast Culture on Intestinal Immune Indicators of Laying Hens Fed Diets with Different Protein Levels. (**A**) PAS-stained sections for counting goblet cells in the jejunum (400×). (**B**) The ratio of goblet cells to 100 epithelial cells on each jejunal villus. (**C**) Jejunal immune indicators, including secretory immunoglobulin A (sIgA), immunoglobulin G (IgG), and immunoglobulin M (IgM). (**D**) Detection results of jejunal inflammatory factors, including tumor necrosis factor-α (TNF-α) and interferon-γ (IFN-γ). Data are expressed as least-squares means and standard errors (*n* = 7). Different letters indicate significant differences (*p* < 0.05), while the same letters indicate no significant difference between groups.

The effects of YC on intestinal immune indicators in hens fed diets with different protein levels are shown in Figure 2. Compared with the NP group, the contents of IgG and IgM in the jejunal mucosa of the LCP group were significantly decreased (*p* < 0.05). Reducing dietary protein also tended to increase IFN-γ content in the jejunal mucosa (*p* = 0.070). Compared with the 0% YC group, 1% YC significantly increased the goblet cell percentage, increased IgG and IgM levels, and reduced TNF-α levels (*p* < 0.05). CP and YC interacted to affect sIgA and IFN-γ contents (*p* < 0.05). Subsequent simple effects analysis showed that relative to the NP-0 group, sIgA content was significantly higher in the LCP-0 group (*p* < 0.05). Compared with LCP-0, sIgA content was significantly lower in the LCP-YC group (*p* < 0.05), returning to a level equivalent to that in the NP-0 group. Compared with LCP-0, IFN-γ content was also significantly lower in the LCP-YC group (*p* < 0.05).

### 3.7. Cecal Microbial Structure and Composition

**Figure 3:**
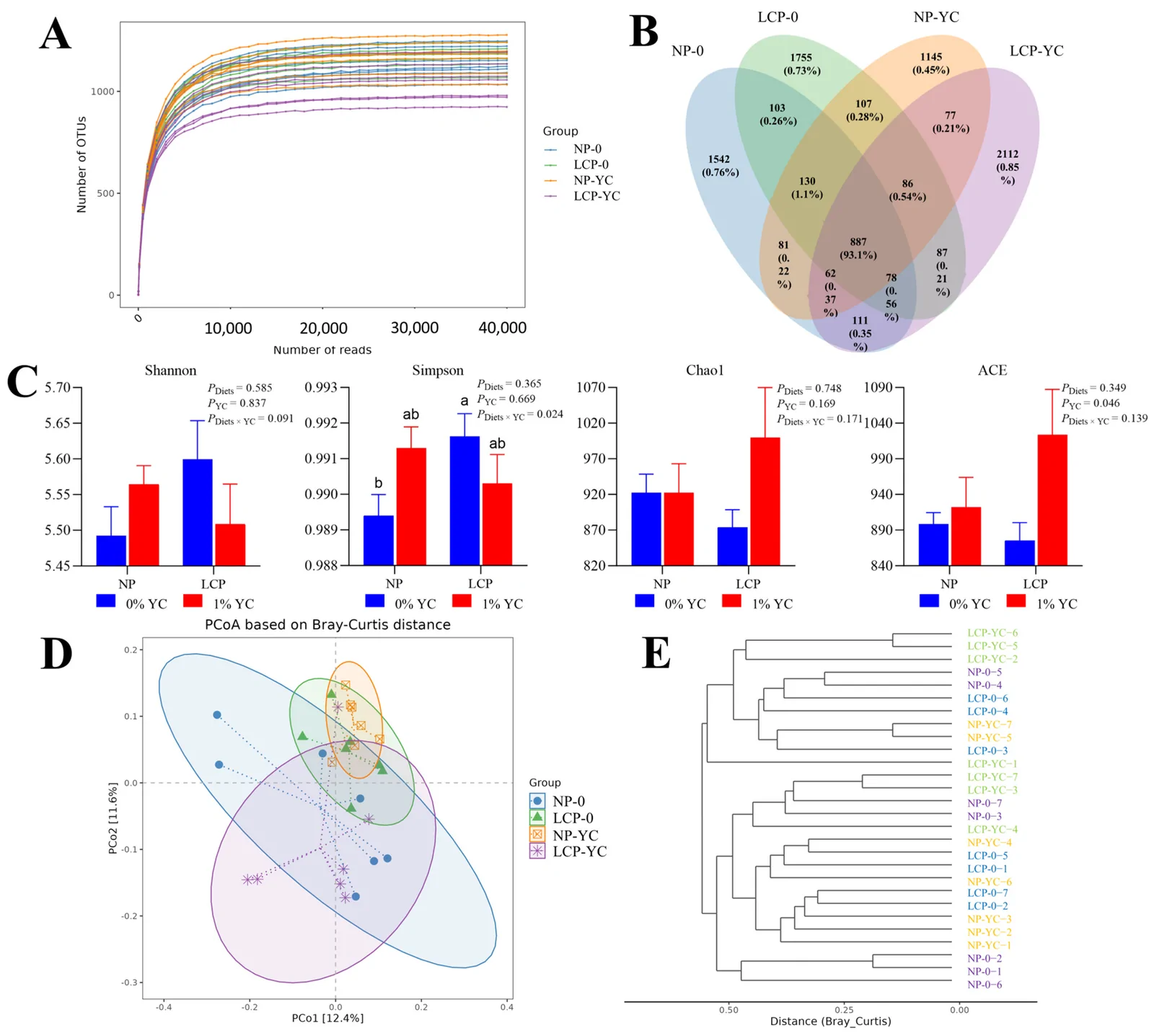
Effects of Yeast Culture on Intestinal Microbiota Diversity of Laying Hens Fed Diets with Different Protein Levels. (**A**) Rarefaction curve. Each line represents an individual replicate (*n* = 7 per treatment group), and line colors indicate the four treatment groups. (**B**) Venn diagram for bacterial OTUs. Minor discrepancies in the sum of percentages are due to rounding. (**C**) Comparison of α-diversity indices, including Chao1, ACE, Shannon, and Simpson. (**D**) Principal coordinate analysis (PCoA) based on the Bray–Curtis dissimilarity matrix was performed to visualize microbial β-diversity. (**E**) Hierarchical clustering tree analysis. Data are expressed as least-squares means and standard errors (*n* = 7). Different letters indicate significant differences (*p* < 0.05), while the same letters indicate no significant difference between groups.

To examine the effects of YC on the gut microbiota of laying hens fed diets with different protein levels, cecal contents were collected for 16S rRNA sequencing analysis. As sequencing depth increased, the species rarefaction curve gradually flattened (Figure 3A), indicating that the sequencing depth had essentially covered the vast majority of species in the samples and could accurately reflect the species diversity of the microbiota in each group. Venn diagram community analysis showed (Figure 3B) that the NP-0 group, LCP-0 group, NP-YC group, and LCP-YC group contained 1542, 1755, 1145, and 2112 unique OTUs, respectively. Alpha diversity analysis indicated (Figure 3C) that YC supplementation significantly increased the ACE index (*p* < 0.05), and dietary CP level and YC interacted to affect the Simpson index (*p* < 0.05). Principal coordinate analysis (PCoA) further showed (Figure 3D) that PCo1 and PCo2 explained 12.4% and 11.6% of the variation in microbial community β-diversity, respectively. Samples from different treatment groups differed somewhat in their distribution in PCoA space, where the NP-YC and NP-0 sample distributions showed some separation, while the LCP-YC and LCP-0 distributions overlapped. Hierarchical clustering analysis revealed a sample clustering pattern similar to that of PCoA (Figure 3E). The PERMANOVA analysis revealed a significant interaction between CP and YC (*p* < 0.05) (Table 6). No significant heterogeneity in multivariate dispersion was detected among the four treatment groups (PERMDISP: pseudo-F = 2.7216, df = 3, 24, *p* = 0.075), indicating that the significant PERMANOVA interaction was unlikely to be driven by unequal group dispersion.

**Figure 4:**
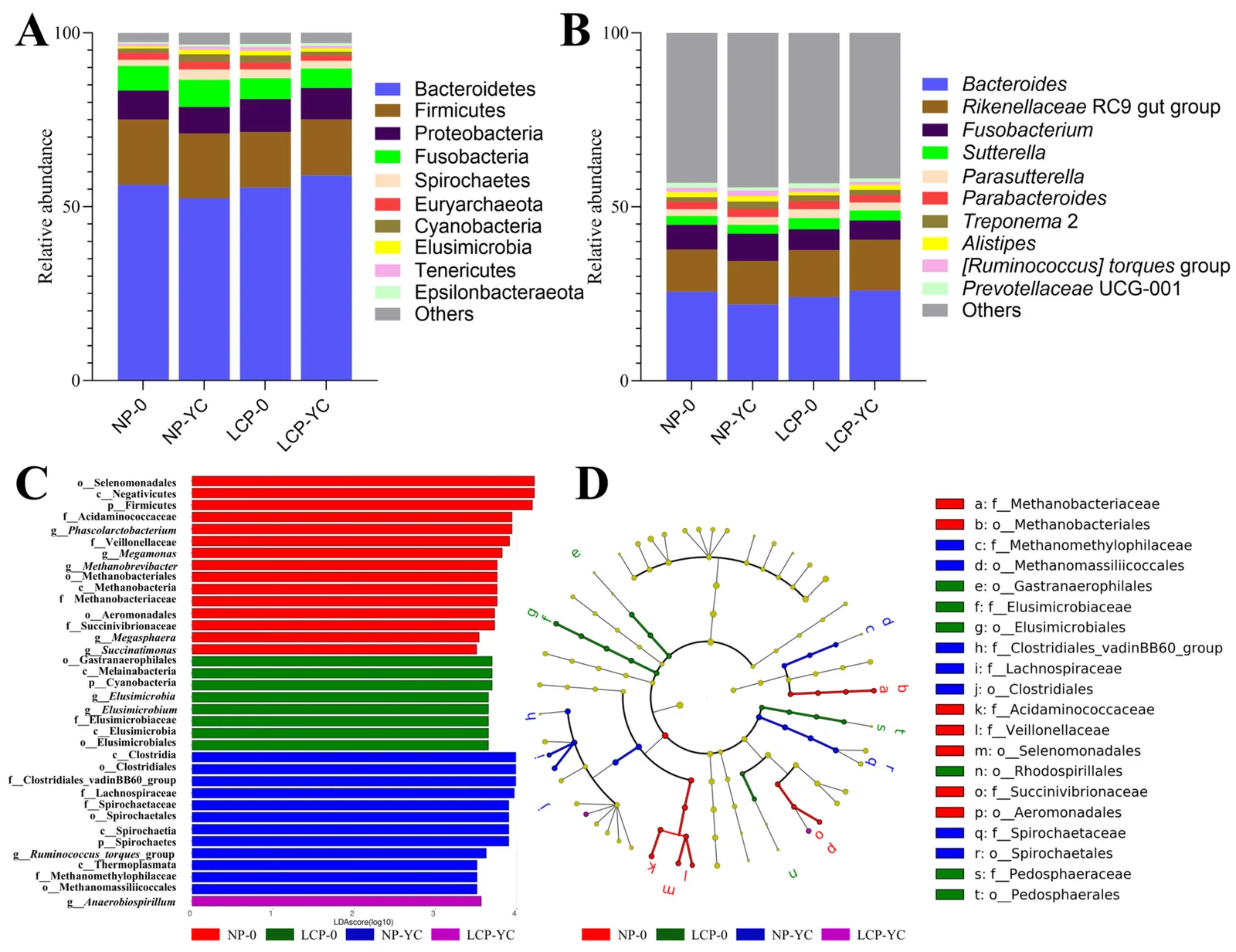
Effects of Yeast Culture on Intestinal Microbial Structure of Laying Hens Fed Diets with Different Protein Levels. (**A**,**B**) Relative abundances of the top 10 microbiota at phylum and genus levels. (**C**,**D**) Linear discriminant analysis effect size (LEfSe) based on phylum- to genus-level (LDA > 3.5), and biomarker taxa are highlighted by colored circles and shaded areas.

Table 7 and Table 8 and Figure 4A, B present the relative abundances of the 10 most abundant microbial phyla and genera. At the phylum level, the main groups were *Bacteroidetes*, Firmicutes, Proteobacteria, Fusobacteria, etc. Gut microbiota phylum composition was similar among groups, but relative abundances differed. Compared with the NP group, the LCP group had significantly lower Firmicutes and significantly higher Proteobacteria abundances (*p* < 0.05). Compared with 0% YC, supplementation with 1% YC tended to increase Spirochaetes (*p* = 0.068). CP and YC interacted to affect the abundance of Spirochaetes, Cyanobacteria, and Elusimicrobia (*p* < 0.05). A subsequent simple effects analysis showed that, compared with the NP-0 group, the abundance of Spirochaetes, Cyanobacteria, and Elusimicrobia was significantly higher in the NP-YC group. At the genus level, the predominant genera were *Bacteroides*, *Rikenellaceae* RC9 gut group, Fusobacterium, etc. Compared with the NP group, the relative abundance of *Rikenellaceae* RC9 gut group in the LCP group tended to increase (*p* = 0.063), while the relative abundance of *[Ruminococcus] torques* group significantly decreased (*p* < 0.05). Compared with the 0% YC group, *Prevotellaceae* UCG-001 relative abundance was significantly lower in the 1% YC group (*p* < 0.05). CP and YC interacted to affect the abundance of *Treponema* 2 and the *[Ruminococcus] torques* group (*p* < 0.05). A subsequent simple effects analysis showed that, compared with the NP-YC group, the LCP-YC group had significantly lower relative abundances of *Treponema* 2 and the *[Ruminococcus] torques* group. LEfSe identified cecal microbial taxa that significantly differed between groups (Figure 4C,D, LDA > 3.5). In the NP-0 group, the families Acidaminococcaceae, Veillonellaceae, Succinivibrionaceae, and the genus *Methanobrevibacter* were significantly enriched. In the LCP-0 group, the class Melainabacteria and the phylum Elusimicrobia were significantly enriched. In the NP-YC group, the abundances of the family Lachnospiraceae, the *[Ruminococcus] torques* group, the *Clostridiales*_vadinBB60_group, and methylotrophic methanogens were significantly increased. In the LCP-YC group, the genus *Anaerobiospirillum* showed the main relative enrichment.

## 4. Discussion

Research suggests that low-protein diets may cause non-essential amino acid deficiencies, thereby reducing poultry performance [20]. In broiler chickens, a 2% reduction in the CP content of the diet resulted in a significant decrease in average daily weight gain [21]. This agrees with our findings. Reducing dietary CP by 1% significantly decreased average egg weight in laying hens. This may reflect a decrease in digestible lysine accompanying the reduction in crude protein. Digestible lysine in the diet has an important effect on egg weight, and increasing the dietary lysine level can increase egg weight [20]. Recent research shows that supplementing low-protein diets with YC can improve production performance [13]. Because YC is rich in prebiotic components such as β-glucans and mannan oligosaccharides, as well as various fermentation metabolites, YC can promote beneficial gut bacterial proliferation, inhibit pathogenic bacterial growth, and maintain gut microbiota homeostasis, thereby improving feed digestibility and utilization and enhancing production performance [5]. In this study, adding YC increased the laying rate. Previous studies have shown that supplementation with 1% YC significantly increased average daily weight gain and improved the feed-to-weight ratio in broiler chickens [22]. To examine how YC improves performance, researchers investigated its intestinal effects. Qiu et al. found that supplementation with 0.1% and 0.2% YC significantly increased VH and the villus-to-crypt ratio in the duodenum and jejunum of laying hens, thereby improving intestinal structure [23]. In addition, supplementation with 0.3% YC increased chymotrypsin and α-amylase activities in duodenal chyme and upregulated genes related to the intestinal barrier and antimicrobial peptides [7]. These findings indicate that YC improves performance by supporting gut health. In our study, CP and YC also interacted to affect laying rate, ADFI, daily egg weight, and qualified egg rate. Subsequent simple effects analysis indicated that, relative to the LCP-0 group, the LCP-YC group had a significantly higher laying rate, significantly greater ADFI, significantly greater daily egg weight, and significantly higher qualified egg rate. Under these experimental conditions, certain indicators responded more favorably to YC in the LCP group. However, the analyzed calcium concentration differed by 0.48 percentage points between LCP-0 and LCP-YC (4.50% vs. 4.98%), corresponding to a 10.7% higher concentration relative to LCP-0. This imbalance may confound the comparison between the two LCP diets. Therefore, the stronger response observed in the LCP-YC group cannot be attributed exclusively to YC or to the CP × YC interaction, and dietary calcium cannot be ruled out as a contributing factor.

Egg yolk color is an important quality indicator closely related to consumer preferences [24]. Studies have shown that yolk color is mainly determined by the carotenoid content and pigmenting efficiency of corn [25]. Therefore, changes in the proportion of corn in the diet can cause significant changes in egg yolk color. In addition, reducing soybean oil content significantly decreases carotenoid deposition in egg yolk [26]. In this study, although the LCP group’s diet contained a higher proportion of corn, the reduced soybean oil content explains the decline in yolk color observed in this group. After dietary YC supplementation, yolk color at week 12 improved numerically, but the difference was not statistically significant. One possible explanation is that YC may improve intestinal health, thereby increasing carotenoid absorption efficiency, allowing greater pigment deposition in the yolk and improving its color [27]. We observed no effect of YC on the other egg quality indicators.

These findings indicated that YC could improve laying hen performance but did not significantly affect egg quality. To examine the underlying mechanisms, we assessed the effects of YC on serum antioxidant levels in hens fed diets with different protein levels. SOD converts superoxide anions into hydrogen peroxide, which CAT then breaks down into oxygen and water. GSH-Px uses glutathione as a hydrogen donor to catalyze peroxide reduction, converting intracellular reduced glutathione (GSH) to oxidized glutathione (GSSG), thereby protecting cells against oxidative damage [28,29]. PCO is the end product of protein oxidation and indicates the degree of oxidative stress in the body [30]. MDA is the end product of lipid peroxidation and is a key measure of its extent [31]. Under normal physiological conditions, the body maintains a dynamic equilibrium between antioxidant and pro-oxidant substances. When reactive substances such as reactive oxygen species accumulate excessively, exceeding the capacity of the animal’s antioxidant system to clear them, oxidative stress occurs [32]. The capacity of poultry to resist oxidative stress depends on their antioxidant defense system. Previous studies showed that supplementation with 1% YC significantly increases hepatic SOD and GSH-Px activity and T-AOC in catfish, lowers MDA levels, and does not significantly affect CAT activity [33]. In laying hens, supplementation with 0.2% YC significantly increased serum T-AOC [27]. In geese, YC increased muscle CAT and glutathione reductase (GR) activities, with effects depending on the dose [34]. However, in weaned piglets, serum T-AOC and SOD activity decreased linearly as dietary hydrolyzed yeast inclusion increased [35]. These differing findings may reflect differences in yeast product manufacturing processes, culture conditions, preparation techniques, and inclusion levels. In this study, YC supplementation significantly increased serum CAT and GSH-Px activities and reduced PCO levels, in agreement with most studies. Yeast cell wall mannan showed strong scavenging activity against both superoxide anions and hydroxyl radicals [36]. In broiler chickens, 0.2% mannan oligosaccharides significantly increased the meat’s DPPH radical scavenging capacity and reduced lipid peroxide levels [37]. In mice, β-glucan significantly increased serum GSH-Px and SOD activity and reduced MDA levels [38]. These studies suggest that these components in YC may have antioxidant effects. However, these yeast-derived components may differ in composition and bioactivity from those of the tested YC. In this study, CP and YC interacted to affect GSH-Px activity. Compared with the NP-YC group, the LCP-YC group showed significantly increased GSH-Px activity. This indicates that dietary CP levels may influence the increase in serum GSH-Px activity induced by YC.

Pancreatic proteolytic enzymes are important for dietary protein digestion and amino acid utilization. In this study, compared with the NP group, trypsin activity was significantly higher in the LCP group, suggesting that reduced dietary protein may stimulate trypsin activity. YC is thought to promote nutrient utilization by improving the intestinal environment and digestive function. Previous studies have shown that dietary YC can increase chymotrypsin and α-amylase activities in the duodenal chyme of laying hens and improve intestinal barrier-related indicators [7], suggesting that YC may support nutrient digestion and utilization by regulating digestive enzyme activities. Supplementation with 1% YC significantly increased trypsin activity in this study, in agreement with previous findings. However, YC also decreased chymotrypsin activity, indicating that its effects differ among pancreatic proteolytic enzymes. Furthermore, this study found an interactive effect between CP and YC on trypsin activity, and simple effects analysis showed that trypsin activity in the LCP-YC group was significantly higher than that in the NP-YC group. These results may indicate that, in a low-protein diet, YC may be more effective at increasing trypsin activity. However, there were also differences in the calcium concentrations measured in the LCP-0 and LCP-YC diets, which may be one of the reasons for the differences in trypsin activity. Therefore, within the scope of this study, it was not possible to distinguish between the respective contributions of dietary protein levels and calcium. Nevertheless, previous studies have shown that functional feed additives may have a greater role when nutrient supply is limited [39]. Finally, although higher trypsin activity may support proteolysis, protein or amino acid digestibility was not directly measured in this study; therefore, these findings do not yet establish that YC improved actual protein digestibility and utilization efficiency in the low-protein diet. Future studies could measure ileal amino acid digestibility, nitrogen retention, and expression of genes related to protein digestion to further test the potential synergistic interaction between YC and dietary protein levels.

We also measured jejunal VH, CD, and the V/C ratio. The results showed that dietary CP levels and YC significantly affected jejunal morphology in laying hens. Compared with the NP group, the LCP group had significantly lower jejunal VH and V/C ratio, suggesting that insufficient protein may limit intestinal epithelial cell proliferation, thereby restricting villus development. This agrees with earlier studies. Reduced dietary protein intake impairs intestinal barrier function, which subsequently reduces intestinal absorption area and compromises overall health [40,41]. YC supplementation significantly increased VH, indicating a beneficial effect on intestinal structural integrity. Previous studies show that YC is rich in β-glucans and yeast proteins, which can improve the intestinal microenvironment by stimulating intestinal epithelial cell growth, regulating the gut microbiota, and enhancing immune responses [42]. In this study, CP and YC interacted to affect jejunal VH (*p* < 0.05). Subsequent simple effects analysis showed that, relative to the LCP-0 group, VH was significantly higher in the LCP-YC group (*p* < 0.05). Similar findings have been reported previously. In diets with reduced energy and protein levels, functional additives can increase jejunal VH and V/C, suggesting that their effects are easier to detect under nutritional restriction [43].

Mucus secreted by intestinal goblet cells contributes to digestion and protects tissue structure. The pro-inflammatory cytokine TNF-α acts at the upstream initiation stage of the inflammatory cascade, whereas IFN-γ can directly act on viruses, parasites, and other pathogens. IgG and IgM are antibodies secreted following B cell activation. Studies have shown that reducing the CP level in the diet increases the levels of the pro-inflammatory cytokines interleukin-1β (**IL-1β**) and interleukin-8 (**IL-8**) in piglet serum, although the patterns of change vary across different growth stages [44]. Bavarsadi et al. reported that reducing dietary CP increases the proportion of eosinophils and decreases the proportion of lymphocytes in laying hen serum, adversely affecting immunity [45]. In this study, reducing dietary CP significantly decreased jejunal mucosal IgG and IgM levels. This may reflect a weaker immune response caused by insufficient amino acid supply and intestinal villus damage resulting from reduced dietary CP [46,47]. Studies have shown that yeast cell wall polysaccharides can suppress harmful bacterial proliferation and abnormal cecal fermentation induced by *Eimeria* infection [48]. Supplementing the diet with 1% yeast cell wall polysaccharides increases serum IgM levels in laying hens and suppresses the excessive expression of pro-inflammatory factors such as TNF-α and IFN-γ in the ileal mucosa [49]. Live yeast and MOS also alleviate *Escherichia coli*-induced intestinal damage. One study found that live yeast and MOS can reduce *E. coli*-induced intestinal inflammation by attenuating the upregulation of *Toll-like receptor 4* (***TLR4***), *nuclear factor-κB* (***NF-κB***), and *IL-1β* gene expression in the ileum [50]. Supplementing laying breeder diets with β-glucan significantly downregulated *TLR4* and *IL-6* mRNA levels in the spleen [51]. In this study, the addition of YC significantly increased the percentage of villus goblet cells in the jejunum, as well as IgG and IgM levels, while reducing TNF-α levels. More goblet cells promote intestinal mucus layer secretion and regulate colonic microbiota structure [52]. Most studies have confirmed that yeast cells and related products can enhance the poultry immune response [51,53]. In addition, we observed an interaction between dietary protein levels and YC affecting jejunal mucosal sIgA and IFN-γ levels. Specifically, adding YC to a low-protein diet restored sIgA to normal levels and significantly reduced IFN-γ. Research has shown that adding yeast glycoproteins to low-fishmeal diets for largemouth bass upregulates the gene expression of anti-inflammatory factors in the intestine and downregulates the gene expression of pro-inflammatory factors [54]. While this agrees with our findings, some studies have reported that adding *Saccharomyces cerevisiae* fermentation products to layer diets significantly increases jejunal IFN-γ levels [55]. These differing results may reflect intestinal stress caused by the low-protein diet. Under intestinal stress caused by the low-protein diet, YC may preferentially suppress the increase in intestinal IFN-γ, thereby reducing potential inflammation and helping maintain immune homeostasis, whereas under non-stressed conditions YC may exert different or even opposite effects on IFN-γ depending on the basal diet and animal physiological state.

This study shows that the addition of YC significantly increased the ACE index. This indicates that YC increased microbial community species richness, in agreement with previous studies. Researchers suggest that YC can benefit the gut microbiota by supplying fermentable metabolites, vitamins, and functional cell wall components [56]. The Simpson index also showed an interaction between CP levels and YC, suggesting a possible synergistic or compensatory mechanism between CP levels and YC. Further analysis of microbial community differences showed that the NP-0 group harbored taxa associated with propionate and succinate metabolism, such as Acidaminococcaceae and Veillonellaceae, and had community characteristics related to carbon-source fermentation and methanogenesis alongside a relatively high abundance of the hydrogenotrophic methanogenic archaeon *Methanobrevibacter* [57]. After YC supplementation, the NP-YC group showed a marked shift toward enrichment of Lachnospiraceae, the *Ruminococcus*_*torques*_group, and the *Clostridiales*_vadinBB60_group within the order Clostridiales, while methylotrophic methanogen relative abundance also changed. However, based solely on changes in the relative abundances of taxonomic groups, it cannot yet be determined whether microbial activity was enhanced or whether SCFA metabolic flux increased. Under low-protein conditions, LCP-0 community composition was characterized by oligotrophy-tolerant taxa represented by the class Melainabacteria. After YC supplementation, only *Anaerobiospirillum* relative abundance increased in the LCP-YC group, without the marked community shifts seen in the NP-YC group. This may be attributed to the fact that under nitrogen limitation, microbial anabolic metabolism is constrained, and the additional carbon sources provided by the YC may not be readily utilized by a broader range of microbial taxa [58]. Consequently, its effects mainly appeared as changes in the relative abundance of a few oligotrophic obligate anaerobes. However, these proposed explanations still require verification through microbial functional gene measurements, metabolomic analyses, or measurements of metabolites such as SCFAs.

## 5. Conclusions

In summary, under these experimental conditions, dietary YC supplementation increased the laying rate, serum CAT and GSH-Px activities, pancreatic trypsin activity, jejunal villus height, goblet cell percentage, and IgG and IgM levels, while reducing PCO, pancreatic chymotrypsin activity, and jejunal TNF-α levels. Significant CP × YC interactions were observed for several indicators, and some responses to YC were more pronounced in the LCP group; however, these findings should be interpreted cautiously because the LCP-0 and LCP-YC diets also differed in analyzed calcium. YC did not significantly affect egg quality. PERMANOVA revealed a significant CP × YC interaction in cecal microbial community structure but no significant YC main effect. This study has several limitations, including differences in digestible amino acid concentrations, the evaluation of only one YC supplementation level, and the absence of direct measurements of microbial functional potential, functional gene expression, or metabolic activity.

## Figures and Tables

**Table 1:** Diet Composition and Nutritional Levels (Air-Dry Basis).

Items	NP-0	NP-YC	LCP-0	LCP-YC
Ingredients				
Corn	62.36	61.71	65.66	64.97
43% Soybean Meal	22.55	22.15	19.65	19.26
Soybean Oil	0.60	0.65	0.19	0.26
Corn Gluten Meal	0.90	0.90	0.90	0.90
DDGS	1.80	1.80	1.80	1.80
Limestone Powder	2.20	2.20	2.21	2.22
Limestone Granules	7.57	7.57	7.57	7.57
Calcium Hydrophosphate	1.00	1.00	1.00	1.00
L-Lysine Sulfate	0.12	0.12	0.12	0.12
DL-Met	0.10	0.10	0.10	0.10
NaCl	0.30	0.30	0.30	0.30
60% Choline Chloride	0.10	0.10	0.10	0.10
Mineral Premix ^1^	0.15	0.15	0.15	0.15
Vitamin Premix ^2^	0.15	0.15	0.15	0.15
Pigment	0.10	0.10	0.10	0.10
Yeast Culture ^3^	0.00	1.00	0.00	1.00
Total	100.00	100.00	100.00	100.00
Calculated Nutrient Content ^4^				
ME (MJ/kg)	11.19	11.19	11.19	11.19
Crude Protein %	15.50 (15.42)	15.50 (15.76)	14.50 (14.27)	14.50 (14.60)
Calcium ^5^ %	4.00 (4.70)	4.00 (4.68)	4.00 (4.50)	4.00 (4.98)
Total Phosphorus %	0.48 (0.42)	0.48 (0.43)	0.48 (0.41)	0.48 (0.45)
D-Lysine %	0.73	0.73	0.67	0.67
D-Methionine %	0.33	0.33	0.32	0.32
D-Threonine %	0.50	0.50	0.47	0.47
D-Tryptophan %	0.14	0.14	0.13	0.13

Note: ^1^ The Mineral Premix provides the following per kilogram of feed: Cu (CuSO_4_) 5 mg; Fe (FeSO_4_) 25 mg; Mn (MnSO_4_) 100 mg; Zn (ZnSO_4_) 60 mg; Se (Na_2_SeO_3_) 0.3 mg; I (KI) 0.5 mg. ^2^ The Vitamin Premix provides the following per kilogram of feed: VA 8400 IU; VD_3_ 3675 IU; VE 69 mg; VK_3_ 2.4 mg; VB_2_ 6.3 mg; VB_6_ 4.2 mg; Niacin 40 mg; VB_1_ 2.4 mg; Biotin 0.11 mg; D-Pantothenic Acid 12 mg; VB_12_ 0.02 mg; Folic Acid 1 mg. ^3^ Nutritional values of the yeast culture were as follows: ME 10.82 MJ/kg; CP 22.00%; Mannan 2.79%; D-Lys 1.03%; D-Met 0.46%; D-Thr 0.85%. ^4^ Values in parentheses were analyzed. ^5^ Each diet sample was analyzed in duplicate. The pooled analytical CV calculated from duplicate determinations across all diets was 1.9% for calcium.

**Table 2:** The Effect of Yeast Culture on Laying Performance of Laying Hens Fed Diets with Different Protein Levels.

Time	Diets	YC	Laying Rate, %	ADFI ^1^, g/hen/d	Average Egg Weight, g	FCR ^2^	Daily Egg Weight ^3^, g/hen/d	Qualified Egg Rate, %
1–6 wk	NP	−	93.03	111.95	61.25	1.97	56.98	92.33
1–6 wk	NP	+	92.87	111.56	61.21	1.96	56.84	91.88
1–6 wk	LCP	−	92.57	111.35	60.82	1.98	56.30	91.69
1–6 wk	LCP	+	94.35	111.83	59.95	1.98	56.55	93.54
7–12 wk	NP	−	91.10	114.26	62.26	2.02	56.75	91.06
7–12 wk	NP	+	91.12	113.23	61.91	2.02	56.12	88.91
7–12 wk	LCP	−	90.95	114.33	61.06	2.06	55.50	89.87
7–12 wk	LCP	+	94.03	115.89	60.69	2.01	57.67	93.01
SEM			0.75	0.41	0.31	0.02	0.43	1.01
Diets	YC							
NP	−		92.06 ^b^	113.11 ^ab^	61.76	1.99	56.86 ^a^	91.69 ^ab^
NP	+		91.99 ^b^	112.40 ^b^	61.56	1.99	56.48 ^ab^	90.39 ^b^
LCP	−		91.76 ^b^	112.84 ^b^	60.94	2.02	55.90 ^b^	90.78 ^b^
LCP	+		94.19 ^a^	113.86 ^a^	60.32	1.99	57.11 ^a^	93.27 ^a^
Time	YC							
1–6 wk	−		92.80	111.65	61.04	1.97	56.64	92.01
1–6 wk	+		93.61	111.70	60.58	1.97	56.70	92.71
7–12 wk	−		91.03	114.30	61.66	2.04	56.12	90.46
7–12 wk	+		92.57	114.57	61.30	2.01	56.89	90.96
Time	Diets							
1–6 wk	NP		92.95	111.76 ^c^	61.23	1.97	56.91	92.11
1–6 wk	LCP		93.46	111.59 ^c^	60.38	1.98	56.43	92.61
7–12 wk	NP		91.11	113.75 ^b^	62.08	2.02	56.43	89.98
7–12 wk	LCP		92.49	115.11 ^a^	60.87	2.04	56.58	91.44
Time								
1–6 wk			93.20 ^a^	111.67 ^b^	60.81 ^b^	1.97 ^b^	56.67	92.36 ^a^
7–12 wk			91.80 ^b^	114.43 ^a^	61.48 ^a^	2.03 ^a^	56.51	90.71 ^b^
Diets								
NP			92.03	112.75	61.66 ^a^	1.99	56.67	91.04
LCP			92.98	113.35	60.63 ^b^	2.01	56.51	92.02
YC								
0			91.91 ^b^	112.98	61.35	2.01	56.38	91.24
1			93.09 ^a^	113.13	60.94	1.99	56.79	91.83
*p*-value								
Time			0.013	<0.001	0.008	<0.001	0.617	0.028
Diets			0.089	0.067	<0.001	0.162	0.602	0.183
YC			0.036	0.631	0.097	0.275	0.198	0.415
Diets × YC			0.027	0.010	0.383	0.255	0.015	0.012
Time × YC			0.503	0.730	0.840	0.333	0.263	0.886
Time × Diets			0.431	0.022	0.462	0.783	0.320	0.515
Time × Diets × YC			0.611	0.189	0.411	0.207	0.061	0.306

Note: ^1^ ADFI = average daily feed intake. ^2^ Feed conversion ratio (FCR) was determined as the ratio of feed intake to egg weight. ^3^ Daily egg weight = total egg weight/(number of laying hens × number of experimental days). ^a^, ^b^, ^c^ Differences in least-squares means within the same column that do not share the same superscript letter are statistically significant (*p* ≤ 0.05). NP: diet crude protein level 15.5%; LCP: diet crude protein level 14.5%; YC: yeast culture. Data represent the least-squares means of 7 replicates per treatment (*n* = 7).

**Table 3:** Effects of Yeast Culture on Egg Quality of Laying Hens Fed Diets with Different Protein Levels.

Items	NP-0	NP-YC	LCP-0	LCP-YC	SEM	Diets		YC, %		*p*-Value		
						NP	LCP	0	1	Diets	YC	Diets × YC
Albumen height, mm	7.04	7.33	7.17	7.09	0.21	7.18	7.13	7.11	7.21	0.805	0.633	0.391
Haugh unit	82.79	84.25	83.33	83.46	1.34	83.52	83.39	83.06	83.85	0.928	0.564	0.629
Yolk color	13.72	13.72	12.58	13.49	0.24	13.72 ^a^	13.04 ^b^	13.15	13.61	0.015	0.094	0.094
Eggshell strength, kg	4.19	4.01	4.45	4.34	0.21	4.10	4.40	4.32	4.18	0.182	0.511	0.870
Eggshell thickness, mm	0.42	0.42	0.43	0.43	0.01	0.42	0.43	0.43	0.43	0.268	1.000	0.779
Egg shape index	1.31	1.31	1.33	1.32	0.01	1.31	1.33	1.32	1.32	0.163	0.396	0.669

Note: ^a^, ^b^ Differences in least-squares means within the same row that do not share the same superscript letter are statistically significant (*p* ≤ 0.05). NP: diet crude protein level 15.5%; LCP: diet crude protein level 14.5%; YC: yeast culture. Data represent the least-squares means of 7 replicates per treatment (*n* = 7).

**Table 4:** Effects of Yeast Culture on Serum Antioxidant Capacity of Laying Hens Fed Diets with Different Protein Levels.

Items	NP-0	NP-YC	LCP-0	LCP-YC	SEM	Diets		YC, %		*p*-Value		
						NP	LCP	0	1	Diets	YC	Diets × YC
T-AOC, mmol/L	0.91	0.82	0.88	0.85	0.08	0.87	0.87	0.90	0.84	0.990	0.478	0.692
MDA, nmol/mL	3.20	2.90	2.67	3.49	0.39	3.05	3.08	2.94	3.19	0.943	0.525	0.178
CAT, U/mL	1.96	4.13	2.14	3.16	0.37	3.05	2.65	2.05 ^b^	3.64 ^a^	0.359	0.001	0.186
SOD, U/mL	295.87	299.22	294.55	312.13	11.13	297.54	303.34	295.21	305.67	0.619	0.372	0.542
GSH-Px, U/mL	2584.07 ^b^	2674.91 ^b^	2503.48 ^b^	2967.45 ^a^	70.14	2629.49	2735.47	2543.77 ^b^	2821.18 ^a^	0.150	0.001	0.015
PCO, nmol/mg	0.56	0.29	0.61	0.29	0.13	0.42	0.45	0.59^a^	0.29 ^b^	0.852	0.046	0.878

Note: T-AOC = total antioxidant capacity; MDA = malondialdehyde; CAT = catalase; SOD = superoxide dismutase; GSH-Px = glutathione peroxidase; PCO = protein carbonyl. ^a^, ^b^ Differences in least-squares means within the same row that do not share the same superscript letter are statistically significant (*p* ≤ 0.05). NP: diet crude protein level 15.5%; LCP: diet crude protein level 14.5%; YC: yeast culture. Data represent the least-squares means of 7 replicates per treatment (*n* = 7).

**Table 5:** Effects of Yeast Culture on Pancreatic Digestive Enzyme Activities of Laying Hens Fed Diets with Different Crude Protein Levels.

Items	NP-0	NP-YC	LCP-0	LCP-YC	SEM	Diets		YC, %		*p*-Value		
						NP	LCP	0	1	Diets	YC	Diets × YC
Amylase, U/mg protein	50.89	44.06	48.57	46.06	5.31	47.47	47.32	49.73	45.06	0.978	0.413	0.703
Lipase, U/g protein	33.46	33.20	32.65	35.72	2.13	33.33	34.18	33.05	34.46	0.711	0.541	0.471
Chymotrypsin, U/mg protein	1.50	0.86	1.76	0.95	0.14	1.18	1.36	1.63 ^a^	0.90 ^b^	0.268	<0.001	0.588
Trypsin, U/mg protein	182.43 ^c^	346.03 ^b^	259.16 ^c^	535.51 ^a^	26.14	264.23 ^b^	397.33 ^a^	220.79 ^b^	440.77 ^a^	<0.001	<0.001	0.044

Note: ^a^, ^b^, ^c^ Differences in least-squares means within the same row that do not share the same superscript letter are statistically significant (*p* ≤ 0.05). NP: diet crude protein level 15.5%; LCP: diet crude protein level 14.5%; YC: yeast culture. Data represent the least-squares means of 7 replicates per treatment (*n* = 7).

**Table 6:** Two-Way PERMANOVA for the Effects of Dietary Protein Level, Yeast Culture, and Their Interaction on Intestinal Microbiota Structure.

Source	df	Sum of Squares	R^2^	Pseudo-F	*p*-Value
CP	1	0.1240	0.0491	1.4268	0.052
YC	1	0.0988	0.0391	1.1359	0.260
CP × YC	1	0.2165	0.0857	2.4898	0.001
Residual	24	2.0867	0.8261		
Total	27	2.5260	1.0000		

Note: PERMANOVA was performed on Bray–Curtis distances with 999 permutations. CP: diet crude protein level; YC: yeast culture.

**Table 7:** Comparative Analysis of Relative Abundance of Dominant Microbiota at Phylum Level %.

Items	NP-0	NP-YC	LCP-0	LCP-YC	SEM	Diets		YC, %		*p*-Value		
						NP	LCP	0	1	Diets	YC	Diets × YC
*Bacteroidetes*	56.29	52.41	55.45	58.99	1.78	54.35	57.22	55.87	55.70	0.134	0.928	0.056
Firmicutes	18.76	18.64	15.98	16.12	0.83	18.70 ^a^	16.05 ^b^	17.37	17.38	0.015	0.993	0.897
Proteobacteria	8.36	7.62	9.50	9.05	0.61	7.99 ^b^	9.27 ^a^	8.93	8.33	0.045	0.338	0.813
Fusobacteria	7.10	7.83	5.99	5.57	1.19	7.47	5.78	6.55	6.70	0.187	0.901	0.647
Spirochaetes	1.72 ^c^	2.93 ^a^	2.56 ^ab^	2.24 ^bc^	0.23	2.33	2.40	2.14	2.59	0.743	0.068	0.003
Euryarchaeota	2.02	2.26	1.96	1.59	0.30	2.14	1.78	1.99	1.93	0.254	0.837	0.334
Cyanobacteria	1.28 ^bc^	2.15 ^a^	2.07 ^ab^	1.00 ^c^	0.26	1.72	1.53	1.68	1.57	0.505	0.710	0.002
Elusimicrobia	0.62 ^b^	1.29 ^a^	1.35 ^a^	1.01 ^ab^	0.17	0.95	1.18	0.99	1.15	0.195	0.352	0.008
Tenericutes	0.74	0.92	1.10	0.72	0.17	0.83	0.91	0.92	0.82	0.666	0.568	0.124
Epsilonbacteraeota	0.44	0.59	0.88	0.75	0.12	0.52 ^b^	0.81 ^a^	0.66	0.67	0.027	0.948	0.293

Note: ^a^, ^b^, ^c^ Differences in least-squares means within the same row that do not share the same superscript letter are statistically significant (*p* ≤ 0.05). NP: diet crude protein level 15.5%; LCP: diet crude protein level 14.5%; YC: yeast culture. Data represent the least-squares means of 7 replicates per treatment (*n* = 7).

**Table 8:** Comparative Analysis of Relative Abundance of Dominant Microbiota at Genus Level %.

Items	NP-0	NP-YC	LCP-0	LCP-YC	SEM	Diets		YC, %		*p*-Value		
						NP	LCP	0	1	Diets	YC	Diets × YC
*Bacteroides*	25.63	21.94	24.02	25.93	1.52	23.78	24.98	24.83	23.93	0.474	0.588	0.100
*Rikenellaceae* RC9 gut group	12.09	12.49	13.52	14.59	0.84	12.29	14.06	12.81	13.54	0.063	0.426	0.711
*Fusobacterium*	7.10	7.83	5.99	5.57	1.19	7.47	5.78	6.55	6.70	0.187	0.901	0.647
*Sutterella*	2.52	2.56	3.22	2.86	0.43	2.54	3.04	2.87	2.71	0.266	0.718	0.663
*Parasutterella*	1.95	2.25	2.52	2.28	0.19	2.10	2.40	2.23	2.27	0.127	0.859	0.171
*Parabacteroides*	2.14	2.35	2.33	2.16	0.15	2.24	2.24	2.24	2.25	0.996	0.914	0.254
*Treponema* 2	1.34 ^b^	2.09 ^a^	1.67 ^ab^	1.49 ^b^	0.17	1.72	1.58	1.51	1.79	0.457	0.135	0.017
*Alistipes*	1.43	1.57	0.95	1.45	0.17	1.50	1.20	1.19	1.51	0.122	0.109	0.344
*[Ruminococcus] torques* group	1.21 ^ab^	1.67 ^a^	1.13 ^b^	0.86 ^b^	0.15	1.44 ^a^	0.99 ^b^	1.17	1.26	0.009	0.561	0.030
*Prevotellaceae* UCG-001	1.45	0.85	1.43	0.94	0.25	1.15	1.19	1.44 ^a^	0.89 ^b^	0.895	0.048	0.821

Note: ^a^, ^b^ Differences in least-squares means within the same row that do not share the same superscript letter are statistically significant (*p* ≤ 0.05). NP: diet crude protein level 15.5%; LCP: diet crude protein level 14.5%; YC: yeast culture. Data represent the least-squares means of 7 replicates per treatment (*n* = 7).

## Data Availability

The raw data supporting the conclusions of this article will be made available by the authors on request.
